# Genetic variation of the *Plasmodium falciparum* circumsporozoite protein in parasite isolates from Homabay County in Kenya

**DOI:** 10.3389/fpara.2024.1346017

**Published:** 2024-04-08

**Authors:** Michael Maina, Sebastian Musundi, Josiah Kuja, Harrison Waweru, Daniel Kiboi, Bernard N. Kanoi, Jesse Gitaka

**Affiliations:** ^1^ Centre for Malaria Elimination, Institute of Tropical Medicine, Mount Kenya University, Thika, Kenya; ^2^ Department of Biochemistry, Jomo Kenyatta University of Agriculture and Technology, Nairobi, Kenya

**Keywords:** circumsporozoite protein, vaccines, genetic diversity, *Plasmodium falciparum*, malaria

## Abstract

The *Plasmodium falciparum* Circumsporozoite Protein (PfCSP) has been used in developing the RTS,S, and R21 malaria vaccines. However, genetic polymorphisms within *Pfcsp* compromise the effectiveness of the vaccine. Thus, it is essential to continuously assess the genetic diversity of *Pfcsp*, especially when deploying it across different geographical regions. In this study, we assessed the genetic diversity of the *Pfcsp* on isolates from Homabay County, a malaria-endemic region in western Kenya, and compared it against other isolates from Kenya. We extracted DNA from 27 microscopically confirmed *P. falciparum* positive samples and conducted Illumina sequencing to generate paired-end short reads. The sequences were then mapped to the Pf3D7 reference genome, and genetic variation was analyzed using bcftools. Additionally, we retrieved isolates from two other malaria-endemic regions in Kenya, Kisumu (n=58) and Kilifi (n=596), from MalariaGEN version 7 and compared their genetic diversity and natural selection. We also evaluated the predicted binding affinities for HLA class I and II supertype alleles for the identified haplotypes using NetMHCpan and NetMHCIIpan. Our results show that the N-terminal of PfCSP was relatively conserved with a notable mutation at A98G across all isolates. The number of NANP repeats varied across the three Kenyan sites within the central repeat region. Furthermore, the C-terminal region showed polymorphism within the Th2R and Th3R regions. Haplotype network analysis of the Kenyan isolates revealed 69 haplotypes, with the 3D7 reference being found in the most prevalent haplotype. When assessing the predicted binding affinities between supertypes in HLA class I and II with the identified haplotypes, we observed stronger predicted binding affinities to multiple haplotypes except for those containing the 3D7 reference. The results suggest the need to take into account the existing changes occurring in *Pfcsp* while developing malaria vaccines.

## 1 Introduction

Malaria poses a huge mortality burden in Sub-Saharan Africa and tropical countries. In 2021, an estimated 241 million infections were reported worldwide, causing about 627,000 deaths (World Health Organization, 2021). This burden was escalated by the COVID-19 pandemic disruptions of existing health systems (World Health Organization, 2021). Despite a declining trend of malaria incidences over the past decade (Rosenthal et al., 2019), pregnant women and children under five still bear the highest burden. *P. falciparum*, the deadliest among the *Plasmodium *spp (Murmu et al., 2021), has a complex life cycle alternating between human and mosquito vectors. This, coupled with a lack of effective vaccines, parasite resistance to the available antimalarials, and mosquito resistance to insecticides, hinder efforts to prevent, control and eliminate malaria (Dimala et al., 2018; Cowell and Winzeler, 2019; Maier et al., 2019).

The WHO rolled out the malaria vaccine initiative to support the fight against malaria (Birkett, 2010; Samuels et al., 2022). It is anticipated that a vaccine, in conjunction with other control measures, can synergize and complement each other to eliminate malaria (Dobanõ et al., 2019). One key target for vaccine development is *P. falciparum* circumsporozoite protein (PfCSP), the major abundant antigen expressed on the surface of sporozoites critical for the invasion of sporozoites in the human liver. Antibodies that target PfCSP have been shown to prevent sporozoite invasion of hepatocytes (Hollingdale et al., 1984; Cohen et al., 2010). The RTS,S (Mosquirix), and R21 are examples of vaccines based on *Pfcsp* sequence. RTS,S and R21 are currently World Health Organization (WHO)-recommended malaria vaccines for widespread use among children living in malaria-endemic countries (Zavala, 2022).

**Figure 1:**
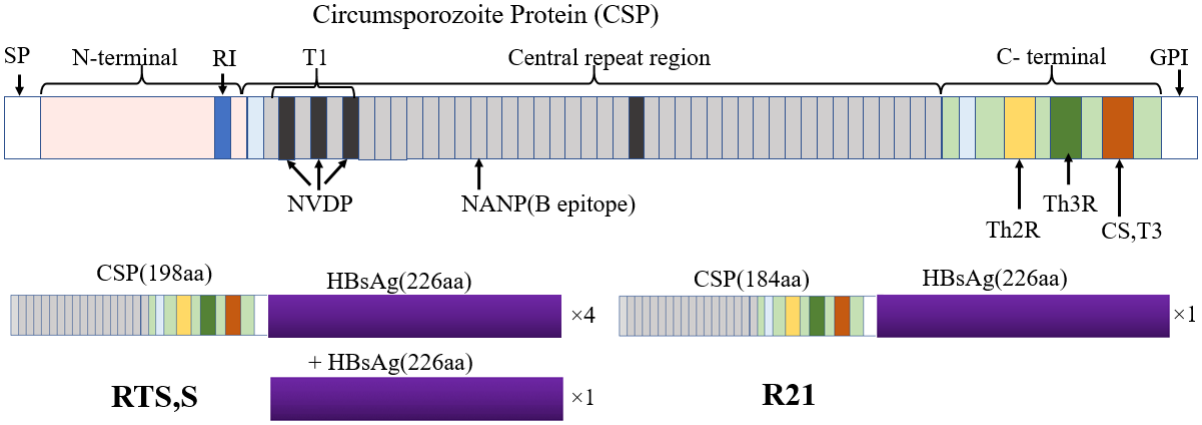
Structure of PfCSP and its associated subunit vaccines RTS,S and R21. PfCSP is divided into three regions; N-terminal region, central repeat region and C-terminal region. PfCSP also contains a signal peptide at the N-terminal end and a glycosylphosphatidylinositol (GPI) at the C-terminal. The N-terminal contains the R1 region, a conserved motif required for sporozoite invasion. The central repeat region contains NANP (Asn-Ala-Asn-Pro) tandem repeats and NVDP (Asn-Val-Asp-Pro). The central repeat region is primarily a B-cell epitope region. The C-terminal region contains three T-cell epitope regions including the polymorphic CD4+ T cell epitope (Th2R), CD8+ T cell (Th3R) and a conserved universal CD4 T cell epitope (CS.T3). The Th3R and CS.T3 region form part of Region II, a thrombospondin like domain. The entire structure of PfCSP is made up of 420 amino acids in the 3D reference. The RTS,S and R21 are subunit vaccines of PfCSP. RTS,S comprises 189 amino acids derived from the last 18 NANP repeats and the C-terminal region with the exception of the GPI anchor fused to four Hepatitis B surface Antigen (HBsAg) (226 amino acids). R21 comprises 184 amino acids derived from NANP repeats and C-terminal region but fused to one HBsAg.

Although RTS,S and R21 are based on *Pfcsp*, slight differences exist structurally. RTS,S/AS01 is a sub-unit vaccine comprising of *Pf*CSP region fused with hepatitis B surface antigen (HBsAg), alongside additional unfused HBsAg assembled into virus-like particles (VLPs) which improve antigen presentation to unique T-cell epitopes (Sinnis and Fidock, 2022). Like RTS,S/AS01, R21/Matrix-M combines a VLP expressing PfCSP-HBsAg fusion protein with a saponin-based adjuvant. Unlike RTS,S, the R21 VLP excludes unfused HBsAg, enabling a 1:1 ratio between PfCSP and Hepatitis B antigen (Collins et al., 2017) (**Figure 1**). Notably, R21 showed at least 75% efficacy over 6 months among Burkina Faso children aged between 5–17 months in phase 2 trials after three rounds of immunization and similar levels of protection even after 1 year of the booster dose (Datoo et al., 2021, 2022). Some reports indicate diminishing effectiveness of vaccines, particularly against *P. falciparum* strains with diverse *Pfcsp* alleles to the region used to make the RTS,S vaccine (Neafsey et al., 2015; Pringle et al., 2018; Laurens, 2019). Thus, understanding the role of genetic diversity of *Pfcsp* has potential implications for vaccine rollout in countries with areas of highly seasonal malaria transmission (Mohamed et al., 2021; Merle et al., 2023).

PfCSP comprises three regions: a conserved N-terminal region, a central repeat region, and a C-terminal region (**Figure 1**). The N-terminal region contains a conserved KLKQP motif required for hepatocyte entry (Sidjanski et al., 1997). The central repeat region is rich in NANP (Asn-Ala-Asn-Pro) tandem repeats and NVDP (Asn-Val-Asp-Pro) and is recognized as the site for antibody-mediated neutralization. The C-terminal region has two polymorphic Th2R and Th3R sub-regions recognized by CD8+ and CD4+ T-cells respectively (Putaporntip et al., 2009; He et al., 2022) (**Figure 1**). Natural selection related to host immunity is hypothesized to be responsible for the polymorphism of these sub-regions (Zheng et al., 2019; Huang et al., 2020). The interaction between PfCSP residues and T-cells is mediated by both HLA class I and II expressed on the surface of antigen-presenting cells (APC), which is essential for T-cell immune response. Polymorphisms observed in the Th2R and Th3R region may directly affect the binding affinity of different PfCSP amino acid residues and T-cells, which can enhance or reduce T-cell immune responses. Understanding vaccine candidates’ genetic and immunological interactions is critical in designing effective vaccines. In this study, we assessed the genetic diversity of *Pfcsp* utilizing paired-end short-read sequences of *P. falciparum* field isolates from Homabay County, a malaria-endemic region in western Kenya. We further compared our results with parasite populations from two malaria-endemic regions in Kenya using *Pfcsp* sequences retrieved from the global MalariaGEN database v7.0. Finally, we predicted the binding affinities of 3D7 reference and non-3D7 variants among the major HLA class I and II supertypes to assess their impact on T-cell recognition.

## 2 Methods

### 2.1 Study site

**Figure 2:**
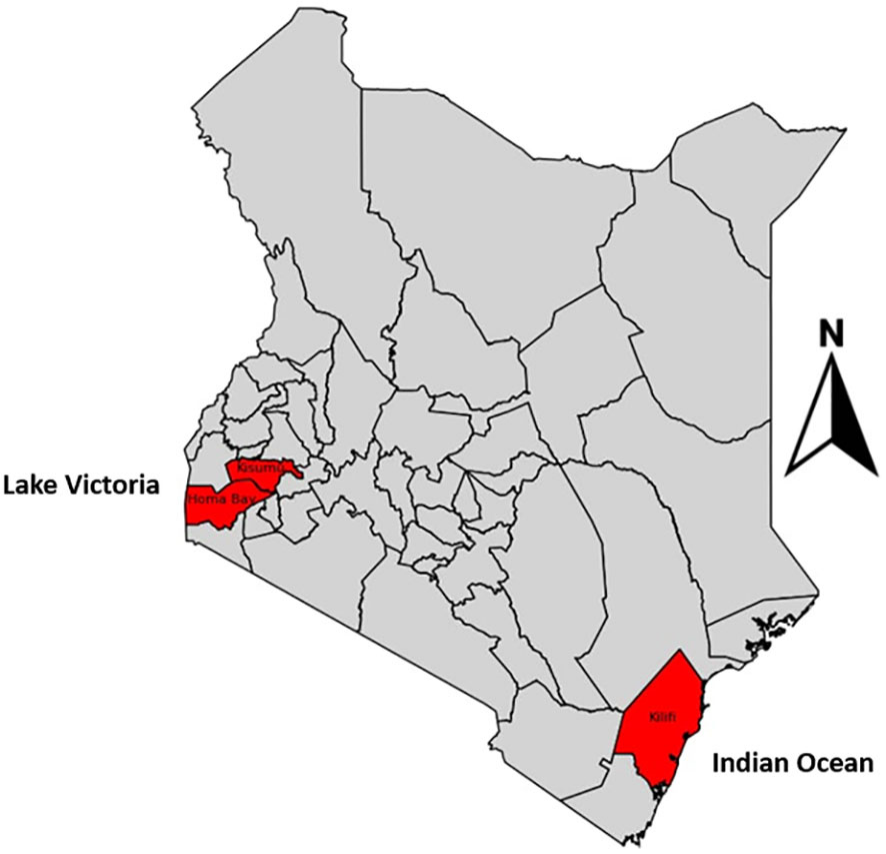
Study sites where sequenced samples were analyzed.

The study was conducted at Homabay Hospital in western Kenya, a malaria-endemic region associated with high malaria transmission intensities. We also included previously sequenced data from MalariaGEN version 7 from Kisumu and Kilifi. Homabay and Kisumu are lake malaria-endemic regions due to their closeness to Lake Victoria, while Kilifi is a coast-endemic region located next to the Indian Ocean (**Figure 2**).

### 2.2 Ethical approval

Approval for the study was obtained from the Mount Kenya University Ethics and Research Review Committee (MKU-ERC; approval P609/10/2014). All participants or guardians provided written consent, and the study adhered to relevant guidelines and regulations. The community was sensitized, and consent was sought before participation, with malaria cases being treated according to national guidelines.

### 2.3 Sample collection, genomic DNA extraction, and sequencing

The samples analyzed in this study were a subset of the larger surveillance studies, which have been extensively described elsewhere (Idris et al., 2016; Gitaka et al., 2017; Osborne et al., 2021). *P. falciparum* species were identified by microscopy following the WHO protocols and confirmed using established nested PCR assays as reported (Osborne et al., 2021). Monoclonal *P. falciparum* parasites obtained from the field were adapted for *in vitro* culture (Osborne et al., 2021). DNA was extracted from short-term parasite cultures (1 month) using the QIAamp DNA mini kit (Qiagen, Valencia, CA). Paired-end sequencing libraries were prepared using Nextera XT DNA library preparation Kit following the manufacturer’s protocol (Illumina, USA). Whole genome sequencing was carried out on Illumina MiSeq technology generating reads of 300bp. These sequences are archived at the DDBJ BioProject, Accession number PRJDB12148 and EBI SRA Project accession PRJEB46180.

### 2.4 Sequence analysis

In this study, the quality of the generated paired-end raw reads was assessed using FastQC. The Plasmodium reference genome (version 63) was retrieved from PlasmoDB and indexed using the Burrows-Wheeler Alignment (BWA) (Li and Durbin, 2009). Subsequently, the paired-end reads were mapped to the indexed Plasmodium genome, generating a SAM file, which was converted, sorted, and indexed into a BAM file. Samtools flagstat was then used to check for the percentage of reads that mapped to the genome. Comprehensive mapping statistics, including sequencing depth and coverage, were also assessed using Samtools (Li et al., 2009). Afterwards, Picard was used to pre-process the generated bam files for variant calling by sorting the order of coordinates, adding read groups, and marking duplicates. All the pre-processed bam files were merged using Samtools merge and were subsequently sorted and indexed. Variant calling analysis was performed using bcftools’ mpileup and call functions. Single nucleotide polymorphisms (SNP) and Indels were then filtered to retrieve variants utilizing a haploid model for those with a base quality score >20 and minimum raw depth >=5. Bcftools view was used to filter biallelic SNPs with a minor allele frequency >0.01 and excluded samples which lacked greater than 10% of the genotypes. Samtools version 1.18 and bcftools consensus was employed to retrieve the CSP protein across all 27 samples (Li et al., 2009). The generated Fasta files were merged, nucleotides translated and aligned using MUSCLE.

We further retrieved the VCF files for chromosome 3 for Kisumu (n=58), a lake endemic region, and Kilifi (n=596), a coast endemic region in Kenya from the MalariaGEN database v7.0. The procedure on how the variant files were generated is previously described (Abdel Hamid et al., 2023). We utilized Gene Analysis Toolkit (GATK) Haplotype caller algorithm to call for SNPs and indels and SelectVariant algorithm to separate SNPs and indels. We also generated merged files containing SNPs and indels using GATK’s MergeVCF algorithm. Subsequently, consensus FASTA files were generated for Kisumu (n=58) and Kilifi (n=596) isolates for *Pfcsp* using Samtools and Bcftools consensus. This involved the replacement of the 3D7 reference nucleotide sequences at specific positions where SNPs and indels were identified. Following this, MEGA suite was employed to translate nucleotides to amino acid residues and aligned each isolate against the *P. falciparum* 3D7 reference using MUSCLE. Metadata for sequence IDs retrieved in MalariaGEN for Kisumu (n=58) and Kilifi (n=596) are provided in **Supplementary Table 1**.

We also aimed to examine population genetic differentiation in the Kilifi samples (n=596) collected across different years (1994–2018). Population genetic differentiation was examined using the fixation index (Fst). Samples collected in individual year and same site were placed under the same population and Fst was calculated using Weir and Cockerham in vcftools. Fst values range from 0 to 1 with high Fst valued indicating a substantial degree of differentiation among the population. The *Fws* metric was also assessed; this evaluates the diversity relation within an individual sample compared to the population by assessing the probability of two random parasites having different alleles at specific positions. *Fws* ranges from 0 to 1 with values ≥ 0.95 representing high inbreeding rates resulting into low host diversity while values *Fws ≤* 0.95 signifying high inbreeding rates and therefore high host diversity. Thus, *Fws* ≥ 0.95 represents monoclonal isolates while *Fws ≤* 0.95 represents polyclonal isolates. For Kisumu (n=58) and Kilifi(n=596) isolates, *Fws* was previously calculated as described (Abdel Hamid et al., 2023).

### 2.5 Genetic diversity of the C-terminal region

Genetic polymorphisms and neutrality tests were assessed the C-terminal region (909–1140 bp) using isolates from Homabay, Kisumu, and Kilifi using DnaSP version 6.12.03 (Rozas et al., 2017). Nucleotide diversity scores for the entire region spanning Th2R and Th3R were obtained and compared across the three sites using a sliding window of 10bp and a step size of 5bp. We also assessed the number of segregating sites(S), number of haplotypes (h), and haplotype diversity (Hd). We also assessed the neutrality of single nucleotide polymorphisms, Tajima D statistical test, Fu and Li’s D and Fu and Li’s F for the entire C-terminal region (Tajima, 1989; Fu and Li, 1993). A web logo plot was also constructed for amino acid residues for Th2R (310–327 aa) and Th3R (352–363 aa) and compared against the 3D7 reference to analyze variant patterns using the WebLogo program. A haplotype network was created to analyze the relationship between the different isolates collected in the three sites in Kenya using the R package geneHapR (Zhang et al., 2023).

### 2.6 *In silico* prediction of the effect of mutations on vaccine efficacy

Since the RTS,S, and R21 vaccines serve as the structural basis for the CSP protein, we sought to examine the impact of individual mutations on CD8+ and CD4+ T-cell responses implicated in CSP immune responses. The Th2R and Th3R regions are associated with CD8+ and CD4+ T-cell responses. As CD4+ T-cells recognize antigens presented through to MHC class II, we utilized NetMHCIIpan 4.0 to predict the binding strength of CSP peptides in the Th3R region utilizing PlasmoDB 3D7 reference sequence and a representative sequence from each of the predicted haplotype (Reynisson et al., 2020). Our selection comprised nine HLA supertypes class II HLA-DRB1 alleles (*0101, *0301, *0401, *0701, *0801, *0901, *1101, *1301, and *1501) that facilitate evaluation of over 95% of the human population HLA supertypes (Reynisson et al., 2020; Kotraiah et al., 2021). We also assessed the impact of variations on the binding affinity of MHCI to CD8+ T-cells lined to Th2R utilizing NetMHCpan 4.1 (Reynisson et al., 2021). For this analysis, we utilized six class I supertype HLA alleles A*0101, A*0201, A*0301, A*2402, B*0702, and B*4403, which cover 90% of the population (Tucker et al., 2021).

## 3 Results

### 3.1 Genetic diversity in the N-terminal region

**Figure 3:**
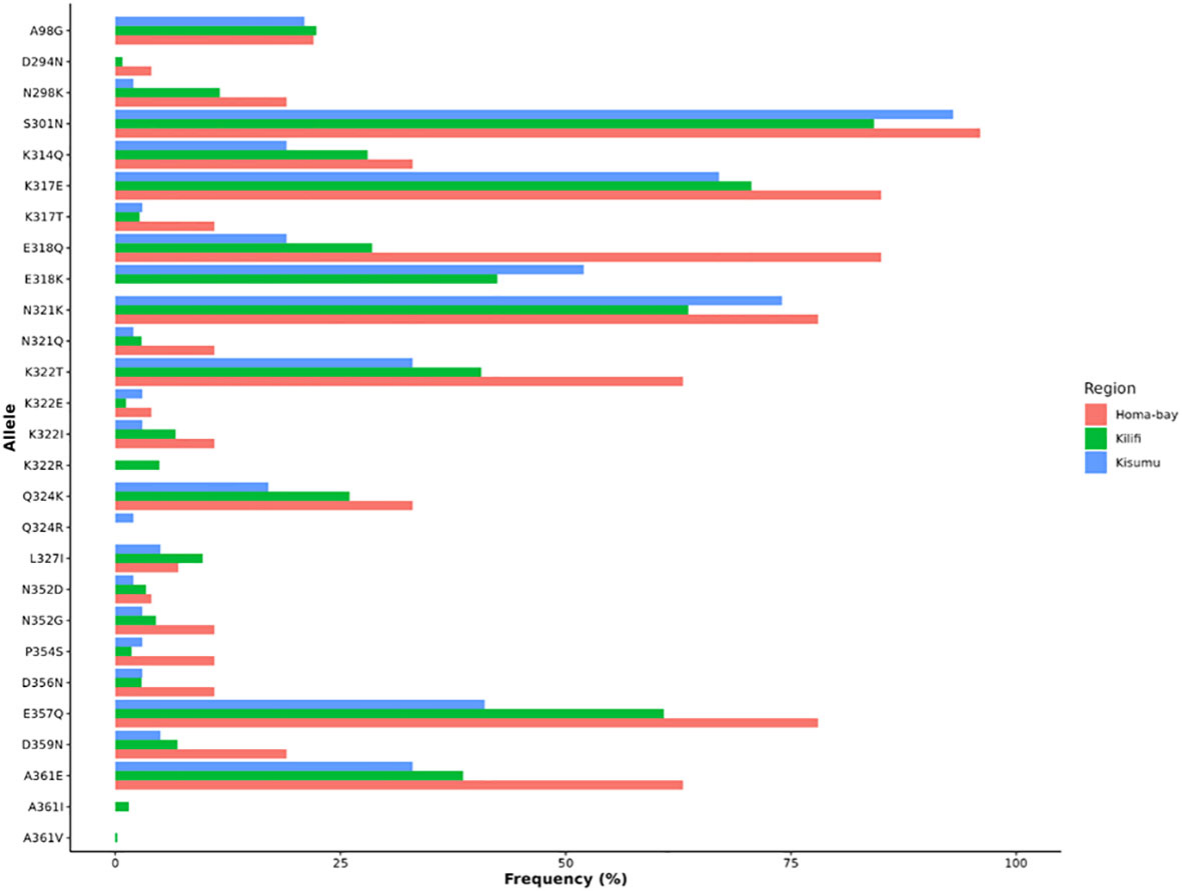
Frequency of mutations found in CSP across Homabay, Kisumu, and Kilifi isolates.

As expected, the N-terminal region remained relatively conserved across Kenyan isolates (**Figure 3**). When compared to the 3D7 reference genome, one variant A98G (6/27, 22.2%), was reported across Homabay isolates. Further comparison with other Kenyan isolates from MalariaGen version 7 revealed the presence of a similar variant at A98G in Kisumu (12/58, 20.7%) and Kilifi (133/596, 22.3%). In addition, low-frequency variants (<1%) were also reported in the Kilifi isolates at Q24A (1/596) and N49S (1/596) (**Figure 3**).

### 3.2 Genetic diversity in the central repeat region

Within the central repeat region, the NANP region remained relatively conserved across Kenyan isolates. Nonetheless, low-frequency variants were identified in Homabay at V126A (1/27), D127N (1/27), and A158V (1/27). In Kilifi variants were found at A186V (2/596), A242V (1/596), D288N (1/596), D294N (5/596). No minor variants were reported across NANP central repeat region in Kisumu isolates. One major variant, S301N, occurred at a high frequency (>80%) across Homabay (26/27, 96.29%), Kisumu (54/58, 93.10%), and Kilifi isolates (502/596, 84.22%) (**Figure 3**). The number of NANP repeats present in the Homabay samples ranged from 33 to 39, with 36 being the most frequent (15/27, 55.55%). In Kisumu, the range was 34–40, with the highest frequencies observed at 39 (29/58, 50%) and 38 (28/58, 48.28%). In Kilifi, the number of NANP repeats ranged from 29 to 41, with 33 being the most frequent (296/596, 49.66%).

### 3.3 Genetic diversity in the C-terminal region across Kenyan samples

**Figure 4:**
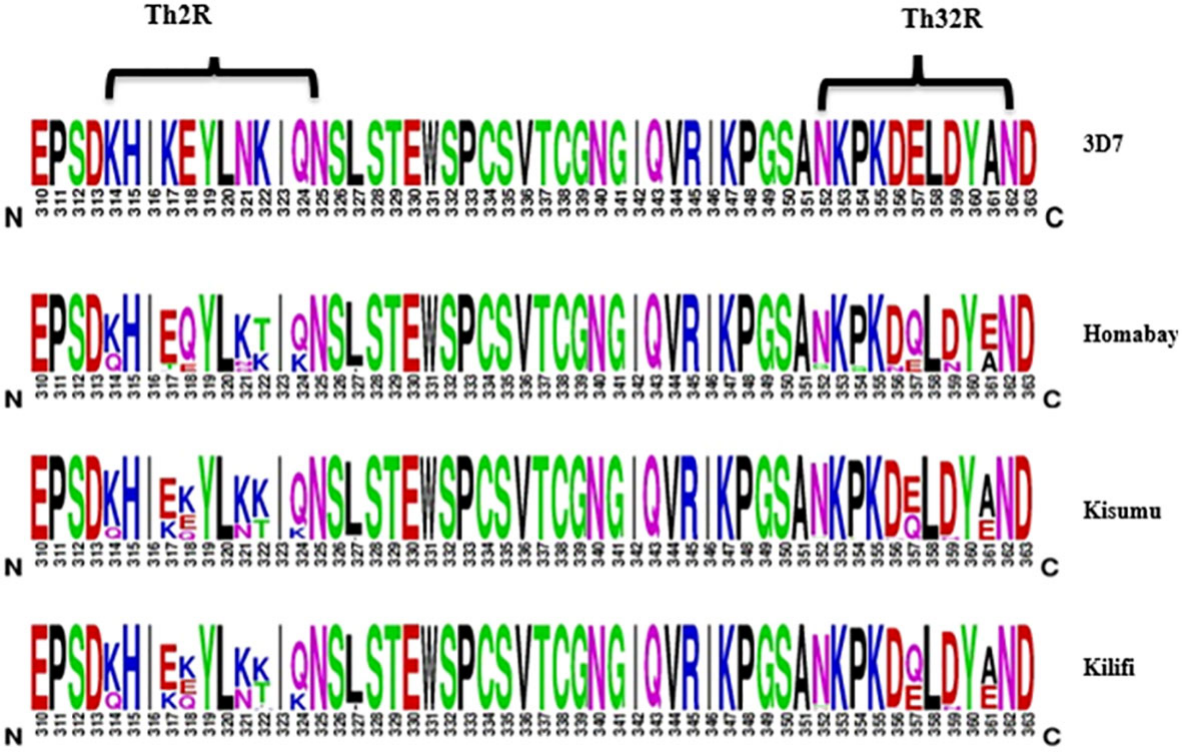
Non-synonymous mutations in the C-terminal region (311–363) of Kenya isolates from Homabay, Kisumu and Kilifi in comparison to the reference 3D7. The height of amino acid indicates their relative frequency at that position. Most amino acid changes were observed between the Th2R and Th3R region. The web logo plot was constructed using the WebLogo program.

Amino acid changes largely occurred in the Th2R and Th3R region of the C-terminal region, as presented in **Figure 4**. A total of four logos are presented, with the first indicating the 3D7 reference strain, while the remaining show isolates from Homabay, Kisumu, and Kilifi. Compared to the 3D7 reference sequence, amino acid changes with high frequency variants (>5%) were observed across 13 positions in the Th2R (K314Q, K317E/T, E318Q/K, N321Q/K, K322T/E/I and Q324K/R, L327I) and Th3R regions (N352D/G, P354S, D356N, E357Q, D359N and A361E/I/V) (**Figure 2**). The changes in K317E, E318K, N321K, and K322T were more common in the Th2R region, while Q324K, E357Q, and A361E in Th3R (**Figure 3**).

**Figure 5:**
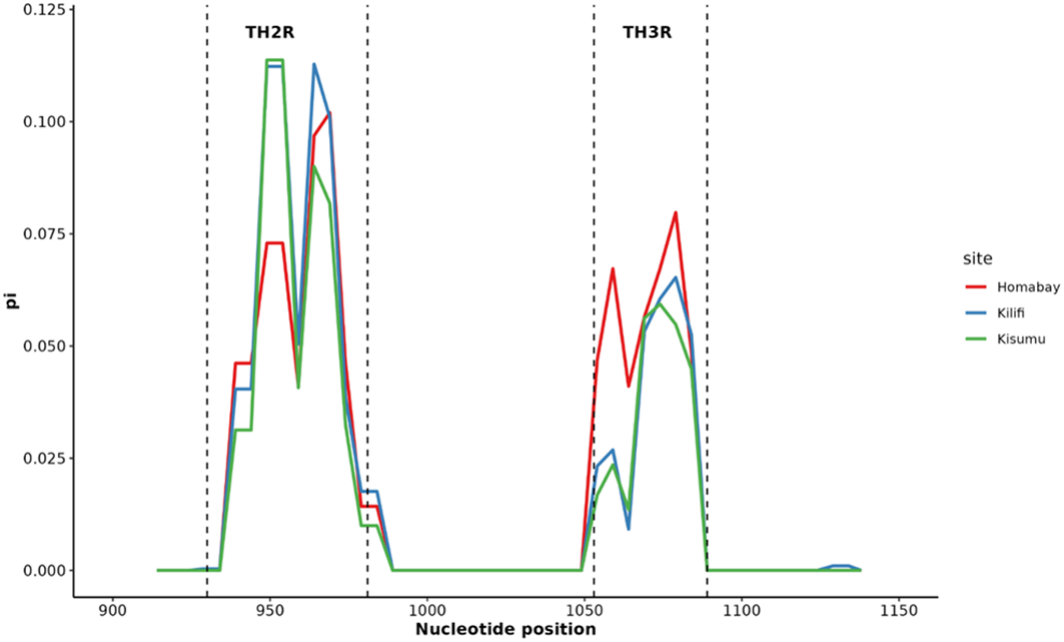
Nucleotide diversity in Th2R and Th3R C-terminal region across Homabay, Kilifi and Kisumu isolates. Nucleotide diversity was calculated using DnaSP with a sliding window of 10 bp with a step size of 5 bp.

Nucleotide diversity of the C-terminal region was calculated using DnaSP for the Homabay, Kisumu and Kilifi isolates using a sliding window of 10 bp and a step size of 5 bp. The nucleotide diversity for Homabay isolates was (Pi: 0.02068), Kilifi (Pi: 0.02019), and Kisumu (Pi: 0.01776). The regions containing the T-cell epitopes in the Th2R and Th3R regions both showed high nucleotide diversity, while the region between Th2R and Th3R was relatively conserved (**Figure 5**). Other parameters associated with natural selection were also evaluated. Population genetic differentiation analysis using Fst using Weir and Cockerham was 0.0047791 for the different population years in Kilifi suggesting a low degree of differentiation for the Kilifi isolates collected over the different years. Both Tajima D (0.306, p >0.1), Fu and Li’s D (1.22904, p>0.1), and Fu and Li’s F (1.10466) were slightly positive but not significant in Homabay samples and across isolates in Kisumu and Kilifi within the C-terminal region which could suggest weak balancing selection (**Table 1**). The haplotype diversity (Hd) for all isolates across the three regions was high (>0.9), indicating high levels of genetic diversity (**Table 1**).

**Table 1:** Nucleotide diversity and tests for neutrality in the C-terminal region of *Pfcsp* in Kenyan isolates.

	n	H	S	Hd ± SD	Tajima D	Fu and Li’s D	Fu and Li’s F
Homabay	27	13	17	0.915 ± 0.00085	0.30688*	1.22904*	1.10466*
Kilifi	596	104	21	0.950 ± 0.00002	0.76834*	0.82698*	0.97593*
Kisumu	58	29	17	0.962 ± 0.00010	0.87513*	0.87513*	0.97593*

(n, Number of isolates; H, Number of haplotypes; S, Number of segregating polymorphic sites; Hd ± SD, Haplotype diversity; *p>0.10).

### 3.4 Haplotype network analysis

**Figure 6:**
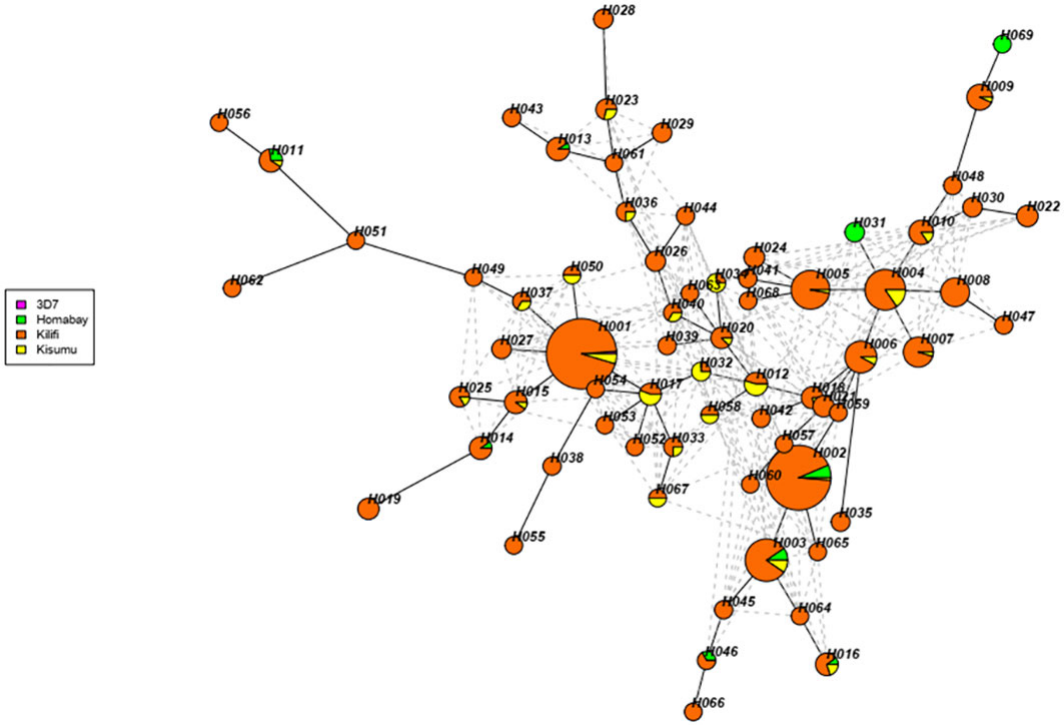
Haplotype network analysis of Kenyan *Pfcsp* C-terminal region. The haplotype network was constructed using the R package gene “geneHapR “. The networks show a total of 69 haplotypes found in 691 isolates and the reference 3D7. The branch length is proportion to the divergence while the size of each node indicates the frequencies of each node. The color of each node represents the different site.

A total of 681 isolates from Homabay (n=27), Kisumu (n=58), and Kilifi (n=596) together with the 3D7, were used to construct the haplotype network. The sequences were clustered into 69 unique haplotypes (H001 to H069). Detailed information for each of the haplotypes is provided in **Supplementary Table 2**. The RTS,S vaccine haplotype, which contains the reference 3D7 strain and denoted as H001, represented 12% (86/681) of all the isolates. Notably haplotype H001 was absent in Homabay isolates (**Figure 6**). Among the haplotypes, H002, H003, H011, and H016 were present across all three sites, while haplotypes H031 and H069 were exclusively present in Homabay isolates. An additional 42 singletons were identified, suggesting that genetic polymorphisms were occurring within the Kenyan isolates.

### 3.5 Interaction between HLA class I and II and *Pfcsp* haplotypes

**Figure 7:**
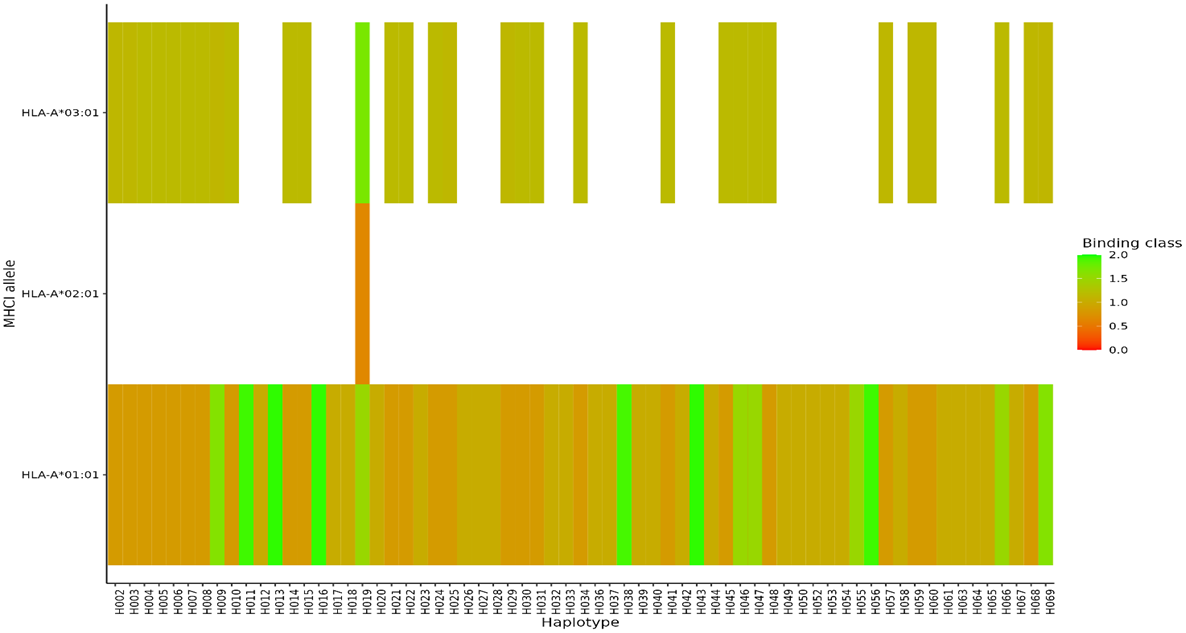
Binding affinities of peptide sequences of each haplotype after proteasomal cleavage of the C-terminal region in MHCI. Peptides classified as strong binders where the % Rank < 0.5% and weak binders where % Rank > 0.5% but less than 2% for MHCI.

**Figure 8:**
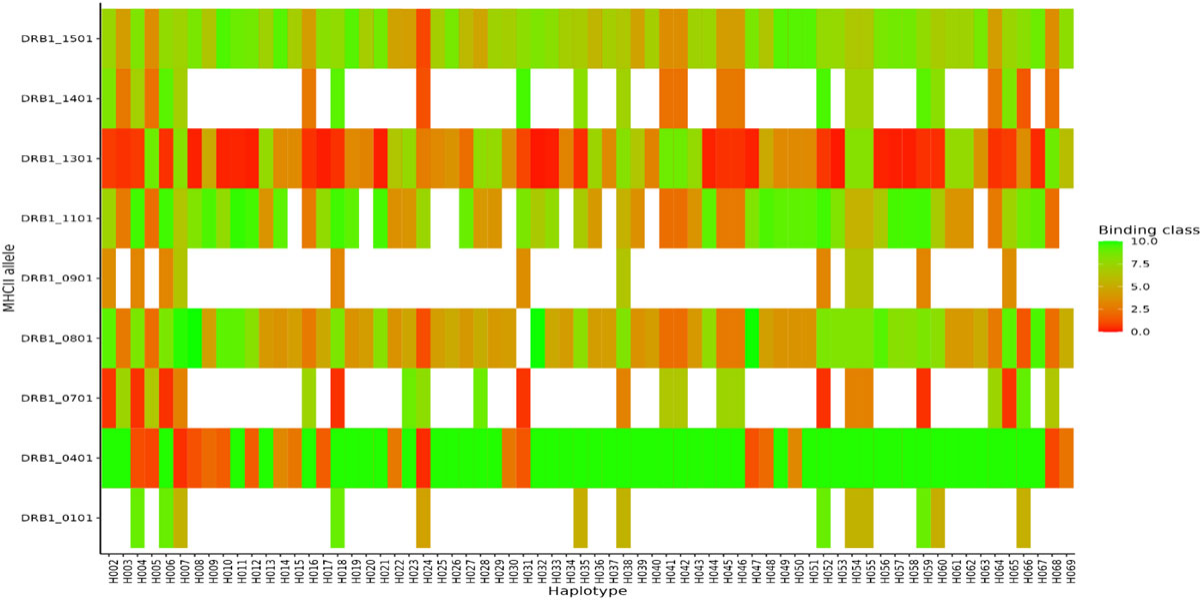
Binding affinities of peptide sequences of each haplotypes after proteasomal cleavage of the C-terminal region in MHCII. Peptides classified as strong binders if the % Rank < 2% and weak binders where % Rank > 2% but less than 10% for MHCII.

For MHC class I, HLA-A alleles 01:01, 02:01, and 03:01 showed higher predicted binding affinities across the 69 haplotypes compared to the other supertype HLA-A alleles 03:01, 24:02, 07:02, and B*44:03 whose predicted binding affinities were below the threshold set for strong or weak binders. Of the three HLA-A alleles which showed both strong and weak binding affinities, allele HLA-A 01:01 showed stronger binding affinities across multiple different haplotypes (**Figure 7**). For HLA class II, DRB_1301 showed stronger binding affinities to multiple haplotypes as compared to the rest of the alleles (**Figure 8**). Nonetheless, HLA class II alleles DRB1_1501, DRB1_1101, DRB1_0801, and DRB1_0401 recognized multiple different HLA alleles with either strong or weak binding activity. The reference strain 3D7 showed weaker binding affinities outside the required thresholds for all HLA class I and II supertypes used in this analysis all haplotypes. Lastly, we did not observe any HLA class I or II allele supertype which showed strong binding affinity to any haplotype.

## 4 Discussion

Vaccine development is a crucial aspect of malaria control and elimination. Various antigens have been identified as potential vaccine candidates for malaria, including RTS,S and R21, both based on *Pfcsp*. A key challenge in malaria vaccine development is the high degree of antigenic diversity due to immune pressure, resulting in different antigenic variants from the reference strain circulating within populations and therefore reducing the effectiveness of current malaria vaccines. This limitation is particularly relevant in endemic malaria regions. It could explain the partial protection reported in RTS,S within the first 12 months and the subsequent waning of the immune response over the next 4 years in children aged between 5 and 17 months (RTS, 2015).

The N-terminal of PfCSP remained relatively conserved across Kenyan isolates, which coincides with other isolates across other regions (Lê et al., 2018; Mohamed et al., 2019; Huang et al., 2020). The N-terminal of CSP binds to heparan sulfate proteoglycans, and cleavage to a conserved region I (KLKQP) by a parasite protease results in to change from being adhesive to invasive to hepatocytes. Specifically, antibodies to the region I found between amino acid ^82^(DNEKLRKPKHKKLKQPADG)^100^ of the N-terminal inhibit the cleavage of CSP and impair invasion both *in vivo* and *in vitro* (Espinosa et al., 2015). In the current results, the non-synonymous mutation A98G was observed in high frequency, as previously reported across most global isolates within region I (Huang et al., 2020). The presence of this non-synonymous mutation suggests the area is under selection pressure and may result in structural changes that have an impact on the sporozoite invasion process in the hepatocyte. Despite this, the conserved motif (KLKQP) involved in heparin sulfate binding remained well conserved across Kenyan isolates. The conserved nature of the N-terminal region, coupled with its key functions, collectively makes it an attractive component of the PfCSP vaccine (Bongfen et al., 2009).

The central repeat region in CSP contains species-specific 4-amino acids NANP/NVDP repeats, which make up the part of the immunodominant B-cell epitopes recognized by neutralizing antibodies. Furthermore, the region has been shown to contribute to sporozoite growth in the mosquito. The different number of tetrapeptide NANP repeats was observed across Kenyan samples, contributing to this region’s polymorphisms. The results are consistent with the number of NANP repeats reported across other Kenyan isolates (Lê et al., 2018) and African isolates (Huang et al., 2020). The differing number of repeats has been shown to affect the PfCSP structure. More specifically, Escalante et al. reported that the stability of type-β PfCSP is increased with the number of repeats (Escalante et al., 2002). The current RTS, S vaccines have 19 NANP tetrapeptide repeats, and to date, there is no direct evidence linking the number of NANP repeats to vaccine efficacy. Nonetheless, targeting regions with repeat regions has been shown to result in less durable antibody responses. Repeat regions such as NANP have been suggested to be decoy regions used by the parasite to prevent the immune system from mounting a strong neutralizing effect.

A high level of polymorphism was observed within the Th2R and Th3R regions of the C-terminal of PfCSP. The Th2R region was more polymorphic compared to the Th3R regions, as previously reported (Amegashie et al., 2020; Dieng et al., 2023). Considering ThR2 and ThR3 regions are involved in the CD4+ and CD8+ T cell responses, the presence of polymorphisms may help the parasite escape the immune pressure from the host, which has a direct impact on the overall efficacy of the vaccine. These results are similar to previous reports, which showed between one and six changes in amino acid occurring within the Th2R and Th3R regions of Zambia, Ghana, and DRC populations (Pringle et al., 2018).

The weak positive Tajima’s D values reported in the C-terminal region of *Pfcsp* across the three different sites in Kenya which could suggest balancing selection primarily due to host immune pressure on the C-terminal. Nonetheless, the positive Tajima’s D values across the three sites were non-significant and therefore inconclusive. Evidence of balancing selection has previously been reported across isolates from Navirongo in Ghana and populations from Malawi (Bailey et al., 2012; Amegashie et al., 2020). The weak positive Tajima’s D values could be further explained by the fact that Homabay, Kisumu, and Kilifi are regarded as malaria endemic regions within Kenya, and therefore, raising the possibility that the variants could be maintained due to their selective advantage, such as conferring better immune response against malaria or being resistant to malaria. The overall values of haplotype diversity for the C-terminal were also high, suggesting a higher level of genetic diversity across Kenyan isolates which is a common feature of most African isolates (Huang et al., 2020). Further analysis of the nucleotide diversity was similar to other global isolates, showing a relatively similar plot composed of high genetic variation within the Th2R and Th3R regions.

Haplotype analysis of the C-terminal region revealed a branched pattern in terms of haplotype diversity. In addition, a total of 42 singletons were identified, suggesting Kenyan isolates show high levels of genetic diversity similar to other African isolates. The 3D7 reference genome used in the development of the RTS,S, and R21 vaccines shared the highest frequency with most isolates from Kisumu and Kilifi but not from Homabay. The genetic heterogeneity might result in different levels of vaccine efficacy, especially in areas where the vaccine haplotype was absent (Pringle et al., 2018). Therefore, consistent monitoring of the existing haplotypes while administering vaccines that target the C-terminal region of CSP will be valuable, especially in high transmission areas. In addition, developing specific individual vaccines that target the most prevalent haplotype in malaria-endemic regions is another solution.

HLA class I and II typically exhibit different binding affinities among diverse populations. Polymorphisms in specific regions recognized by HLA alleles can either provide protection against a variety of pathogens or increase susceptibility to diseases. The strength of binding between antigenic peptides and the groove of the HLA, as well as with T-cell receptor peptides, determines TCR activation, signaling, and cell responsiveness (Baumgaertner et al., 2022). Given that regions Th2R and Th3R are crucial for evoking CD4+ and CD8+ responses, examining their interaction with HLA alleles offers insights into the impact of existing variants on T-cell responses. Our predicted results indicate that multiple haplotypes exhibit better binding affinity compared to the reference strain 3D7 for both HLA class I and II supertypes. These findings align with observations from the evaluation of Ghanian isolates. In that context, the 3D7 reference clearly bound to the HLA class II allele, suggesting that the reference might be binding to additional epitopes present in PfCSP, which were not assessed in this study (Dieng et al., 2023).

The samples used in this study were collected from Homabay in Kenya, one of the malaria-endemic regions in Kenya. The different malaria endemic regions have unique parasite transmission diversities that may continuously impact CSP diversity. To understand the complete CSP diversity in Kenya, it is important to assess mutations across the country continuously. Also, the study only utilized the major HLA class I and II supertypes present globally, and might not provide a representative picture of Kenyan HLA class I and II supertypes since which are are currently not resolved especially among malaria endemic regions. Other factors such as that may influence HLA variation and T-cell responses, such as exposure differences, T-cell receptor variation, and vaccination status, were not examined. Lastly, the sequenced isolates used in analyzing the current data were cultured *in vitro* and therefore, there is also possibility of some mutations being introduced during the culturing process.

## 5 Conclusion

This study provides essential information on the genetic diversity and polymorphism of *Pfcsp* from Kenya. The N-terminal region and central repeat region within the Kenyan isolates remained relatively conserved, while the Th2R and Th3R in the C-terminal region showed high levels of polymorphism. Lastly, the 3D7 reference did not bind weakly or strongly to supertype alleles, although multiple different haplotypes were able to be recognized by the same HLA alleles.

## Data availability statement

The datasets presented in this study can be found in online repositories. The names of the repository/repositories and accession number(s) can be found in the article/**Supplementary Material**.

## Ethics statement

The studies involving humans were approved by Mount Kenya University Ethics and Research Review Committee (MKU-ERC). The studies were conducted in accordance with the local legislation and institutional requirements. Written informed consent for participation in this study was provided by the participants’ legal guardians/next of kin. The animal study was approved by Mount Kenya University Ethics and Research Review Committee (MKU-ERC). The study was conducted in accordance with the local legislation and institutional requirements.

## Author contributions

MM: Conceptualization, Data curation, Formal analysis, Investigation, Methodology, Software, Supervision, Validation, Visualization, Writing – original draft, Writing – review & editing. SM: Data curation, Formal analysis, Investigation, Methodology, Software, Validation, Visualization, Writing – review & editing. JK: Data curation, Formal analysis, Investigation, Methodology, Software, Visualization, Writing – review & editing. HW: Data curation, Formal analysis, Investigation, Methodology, Validation, Visualization, Writing – review & editing. DK: Data curation, Investigation, Supervision, Validation, Visualization, Writing – review & editing. BK: Conceptualization, Data curation, Formal analysis, Investigation, Supervision, Validation, Writing – review & editing. JG: Conceptualization, Data curation, Formal analysis, Funding acquisition, Investigation, Methodology, Project administration, Resources, Supervision, Validation, Writing – review & editing.

## Supplementary material

Supplementary Table 1 Metadata for Homabay, Kisumu and Kilifi isolates retrieved from MalariaGEN version 7.

Supplementary Table 2 Haplotype network analysis dataset showing the classification and frequency of each isolate to their haplotype
